# Laparoscopic resection of an adrenal oncocytic neoplasm: Report of a case and review of the literature

**DOI:** 10.1016/j.ijscr.2020.09.185

**Published:** 2020-09-29

**Authors:** P. St-Amour, R. Djafarrian, T. Zingg, S. La Rosa, N. Demartines, M. Matter

**Affiliations:** a Department of Visceral Surgery, University Hospital of Lausanne (CHUV), Lausanne, Switzerland; b Institute of Pathology, University Hospital (CHUV) and University of Lausanne (UNIL), Lausanne, Switzerland

**Keywords:** AON, adrenocortical oncocytic neoplasm, BMI, body mass index, CK, cytokeratin, CT, computed tomography, EMA, epithelial membrane antigen, ESES, European Society of Endocrine Surgeons, ^18^F-FDG PET-CT, 18F-fluorodesoxyglucose positron emission tomography, HPF, high-power fields, MRI, magnetic resonance imaging, PAX8, paired-box gene 8, SUVmax, maximal standardized uptake value, Adrenalectomy, Adrenal oncocytic neoplasm, Endocrine surgery

## Abstract

**Introduction:**

Oncocytic adrenal neoplasms are rare and mostly benign lesions. Available literature supports indication for a surgical resection, but criteria to predict aggressive behavior are unreliable, thus making decision of surgical approach (laparotomy versus laparoscopy), and extent of resection, difficult to define.

**Presentation of case:**

This is the case of a 46-year-old male, with an incidental finding of a 10 cm asymptomatic tumor in the left adrenal gland identified by MRI, performed in the setting of the initial assessment of liver steatosis. Adrenal hormone levels were in the normal range, thus, a CT-guided needle biopsy was performed and showed an adrenocortical oncocytic neoplasm. A laparoscopic left adrenalectomy was performed sparing the adjacent left kidney. Histological examination of the resected tumor showed a 10 cm oncocytic adrenocortical neoplasm of uncertain malignant potential with negative resection margins. A follow-up MRI was scheduled at six months after surgery, and no recurrence was found.

**Conclusions:**

Although rare, oncocytic neoplasms should be included in the differential diagnosis of adrenal “incidentalomas”. Determination of their malignant potential is difficult in the preoperatory setting. Final diagnosis is based on histological analysis of the whole surgical specimen. Laparoscopic complete excision with negative resection margins is feasible and safe.

## Highlights

• Oncocytic adrenal neoplasms are rare and mostly benign lesions.
• Preoperative determination of malignancy remains difficult.
• Surgical excision planification is based on preoperative investigations.

## 1 Introduction

Adrenocortical oncocytic neoplasms (AONs) are rare tumors, with the most recent systematic review published by Kanitra in 2018 [1], including 140 cases. In total, there are only 227 cases described in the literature until 2019 [2]. Literature review is summarized in Table 1. Oncocytic endocrine and neuroendocrine tumors can also be discovered in other organs including thyroid, parathyroid, pituitary gland, lungs and pancreas [3]. Heterotopic adrenal tissue with oncocytic transformation has also been described [4]. Onconcytic tumor synchronous with tumors of other organs have also been observed (i.e. papillary thyroid cancer) [5]. AONs are more frequent in women and mean age at diagnosis is 47 years (range 27–72 y.o.) [6]. They are more often localised in left adrenal gland, with a well-defined capsule and a diameter of up to 23 cm (mean = 8 cm) [2,6,7].

**Table 1:** Summary of the literature.

Refs.	Authors	Publ. Year	Study type	Nb of cases	Women percent	Laparoscopy percent	Benign/Uncertain/Malign percent^a^	Median tumor size (range) [cm]
[2]	Virarkar et al.	2019	Case report	4	100%	Not defined	Not defined	6 (3.5–8.5)^b^
[24]	Mills et al.	2019	Retrospective	9	22%	11%	0%/0%/100%	19.8 (4.2–28.5)
[20]	Renaudin et al.	2018	Retrospective	43	58%	33%	21%/14%/65%	7.5 (4.5–10.5)
[9]	Peynirci et al.	2018	Retrospective	11	55%	Not defined	64%/36%/0%	5.8 (2.5–13)
[1]	Kanitra et al.	2018	Systematic review (and case report)	141	66%	37%	35%/41%/24%	8 (1.6–28.5)
[13]	Costanzo et al.	2018	Case report (and systematic review)	1	100%	Conversion	Not defined	9.9
[5]	Podetta et al.	2017	Case report	1	100%	0%	100%/0%/0%	8.5
[10]	Ertan et al.	2017	Retrospective	16	50%	Not defined	75%/6%/19%	Not defined
[11]	Sumner et al.	2017	Case report	1	100%	0%	0%/0%/100%	23

Description of the latest systematic review of the literature and cases not included in its study period.

Ref = Reference ; Publ = Publication ; Nb = Number ; percent = percentage.

a According to Lin-Weiss-Bisceglia score, 2004.

b This value refers to mean (range).

The preoperative evaluation of possible malignancy, the optimal surgical approach and the extent of resection (potentially including adjacent organs) remain challenging [1].

The work has been reported in line with the SCARE criteria [8].

## 2 Presentation of case

**Fig. 1:**
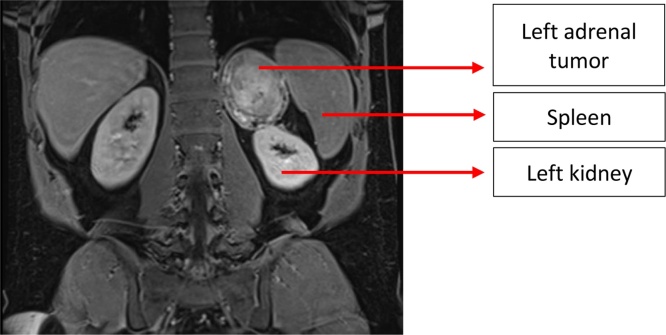
MRI showing the large tumor adjacent (in contact) with the left kidney.

A 46-year-old patient with a non-alcoholic steatohepatitis, stage F1 fibrosis and overweight (BMI 29.86 kg/m^2^) had no oncologic past medical history. He had no relevant family history of endocrine diseases and was on antidepressant medication (Escitalopram). The initial assessment of the hepatopathy with abdominal MRI incidentally revealed a heterogeneous lesion of the left adrenal gland, measuring 62 × 69 mm, with a spontaneous hyperintense T1 signal (Fig. 1). With a history of blunt abdominal trauma a few months earlier, an adrenal hematoma was suspected.

**Fig. 2:**
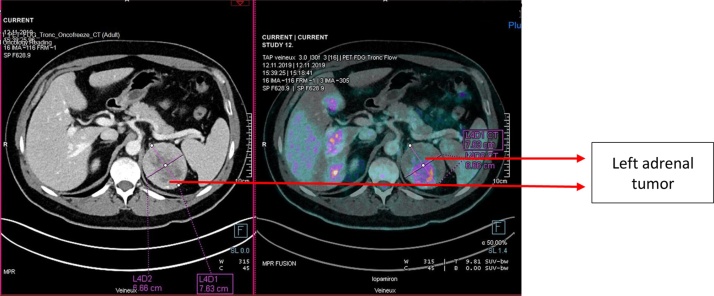
^18^FDG-PET-CT combined with contrast CT-scan showing the hypermetabolic left adrenal tumor, without distant metastases.

**Fig. 3:**
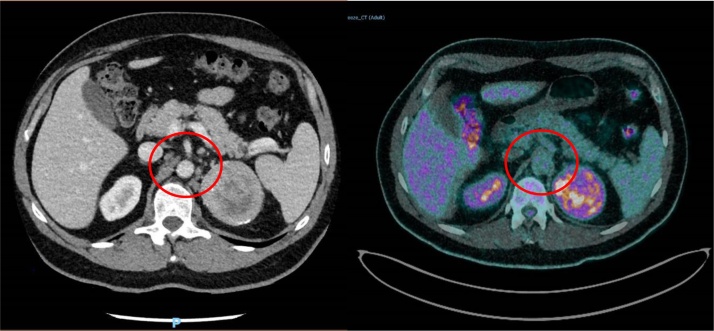
^18^FDG-PET-CT combined with contrast CT-scan showing para-aortic and epigastric not hypermetabolic enlarged lymph nodes.

The patient was asymptomatic, without any hormonal abnormality. An abdominal CT-scan was performed three months later, which confirmed the left adrenal lesion (67 × 79 × 83 mm) with a necrotic center and calcifications, suggesting an adrenal carcinoma. Multiple left-sided retroperitoneal lymph nodes, up to 13 mm in size, were also found. The clinical differential diagnosis included adrenal neoplasm and retroperitoneal sarcoma. The patient underwent ^18^F-FDG PET-CT, revealing a hypermetabolic lesion of 67 × 76 × 85 mm, with a SUVmax of 37. Neither other lesions nor hypermetabolic lymph nodes were found (Fig. 2, Fig. 3).

In order to plan the surgical approach and to determine the need for an en-bloc retroperitoneal resection including the left kidney, a preoperative biopsy of the lesion was performed that concluded to AON.

After a multidisciplinary discussion, a laparoscopic left adrenalectomy (two 12 mm and one 5 mm trocars), with extraction of the tumor through a subcostal mini-laparotomy protected by a plastic bag (operative time: 141 min) was performed. Asymptomatic low plasmatic cortisol level was observed at post-operative day one, and a substitution was prescribed for 3 days. The patient was discharged home at day 4 after surgery. At two-weeks follow-up, he reported a slight dyspnoea. A dedicated CT-scan revealed distal pulmonar embolisms, despite post-operative antithrombotic prophylaxis. He was successfully treated with anticoagulation.

**Fig. 4:**
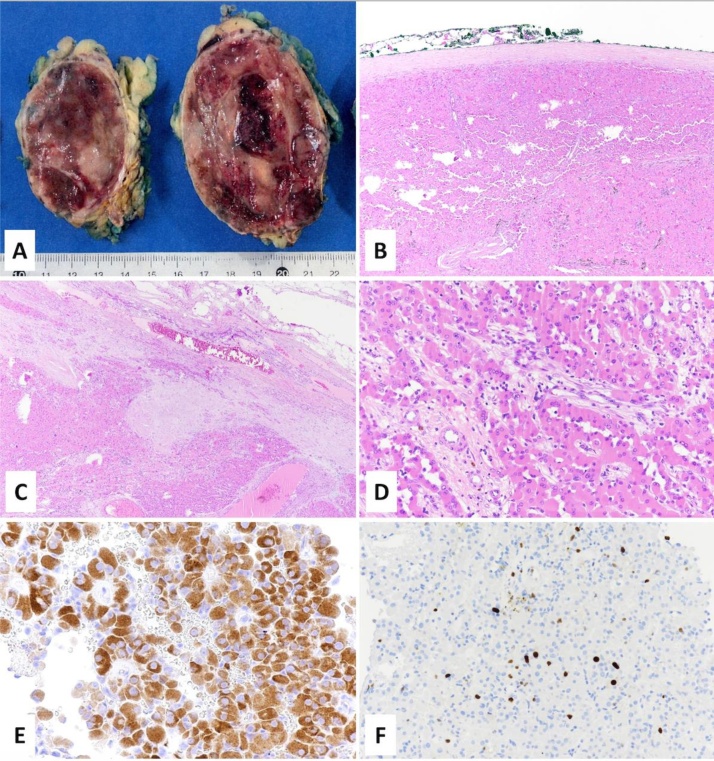
Macroscopically the tumor was well-circumscribed with a red-brown colour (A), delimited by a fibrous capusule (B) which was focally infiltrated (C). Tumor was composed of large cells with abundant eosinophilic cytoplasm (D), positive for melan A (E). The ki67 proliferative index was 3% (F).

Pathology examination showed a well delimitated and encapsulated 10 cm sized neoplasm weighting 325 g. On section, it was red-brown with a central haemorrhagic area (Fig. 4A). Histologically, the tumor showed a solid and trabecular architecture and was composed of large cells with abundant eosinophilic cytoplasm and centrally located nuclei with prominent nucleoli. Less than 5 mitoses per 50 high-power fields (HPF) were observed, without atypical mitotic figures. Neither vascular invasion, sinusoidal invasion, nor necrosis were found. Focal capsular infiltration was observed. Tumor cells were positive for Melan A and negative for EMA, Cytokeratin (CK) 20, CK7, calretinin, chromogranin, inhibin, S100, and PAX8. The Ki67 index was 3% (Fig. 4B–D). The Lin-Weiss-Bisceglia scoring system only included two minor criteria (size and capsular infiltration) and for this reason the neoplasm was diagnosed as AON of uncertain malignant potential [12]. The multidisciplinary tumor board recommended clinical-radiological follow-up with MRI every 6 months. The first scheduled MRI revealed no signs of recurrence.

## 3 Discussion

The challenge is to determine the malignant nature or not of AONs to plan the type of surgical resection. These tumors are rare, usually benign and large tumors. They mainly occur in middle-aged women [13].

AONs are functional in about 17% of cases, and, as for all cases of adrenal tumors management, hormonal status must be assessed [6].

Radiological findings are unspecific. The available literature suggests that a density of less than 10 HU (lipid-rich) on CT scan, without any necrosis or cyst, is more likely associated with benign lesions but the diagnosis and malignancy cannot be determined based on these characteristics [6,14]. Similarly, US, MRI and ^18^F-FDG-PET-CT findings can guide, but not confirm, the malignancy status [15].

Available literature on adrenal “incidentalomas” only recommend preoperative fine-needle aspiration in selected cases, as it may be associated with some complications (risk 0–12%) and can have false negatives. In fact, biopsies should be performed in cases where surgical attitude could be affected by these results (distinction between benign and malignant lesions), or in presence of other known malignant lesions [1,16,17]. These rare tumors should be managed in tertiary reference center for endocrine surgery that follow ESES recommendations [17].

The histological hallmark of AON is the presence of oncocytic cells, which were described in the adrenal glands by Kakimoto et al. in 1986 [18]. The prognostic classification of AONs is based on histological features and includes the following entities: benign AON, AON of uncertain malignant potential and malignant AON [2,12]. Malignancy is found in about 20% of these tumors and can only be determined histologically after surgical resection [6].

The first classification scheme of adrenocortical neoplasms was proposed by Weiss [19]. Since the presence of less than 25% clear cells suggests malignancy, the typical morphology of oncocytic neoplasms is *per se* a criterion of malignancy. For this reason, a revision of this classification was performed by Bisceglia et al. to produce an *ad hoc* scheme especially adapted for oncocytic adrenocortical tumors (Lin-Weiss-Bisceglia criteria) [12,20]. Major criteria of malignancy include high mitotic rate (>5 mitoses × 50HPF), the presence of atypical mitoses and venous invasion. Minor criteria include large size (>10 cm and/or more than 200 g), the presence of necrosis, capsular infiltration, and sinusoidal invasion. In presence of one major criterion, malignancy is confirmed. Moreover, if one to four minor criteria are present, malignancy potential remains uncertain. If none of the criteria is present, the lesion is considered benign [1,6,12]. The immunohistochemical profile can help for the diagnosis, especially on biopsy material. Markers such as Ki-67 and topoisomerase have been proposed to help in determining malignancy [6,7].

In the present case, the malignancy potential could not be determined after multiple imaging and percutaneous biopsy. We decided to perform a laparoscopic resection after multidisciplinary discussion, and final histology concluded to an AON of uncertain malignant potential. Follow-up did not reveal any recurrence.

Concerning adrenal tumors, international guidelines recommend laparoscopic resection for benign lesions and pheochromocytomas. Many authors have shown interest in analysing superiority of laparoscopy over open approach for adrenocortical carcinoma, but literature is still conflicting. At this day, gold standard remains open resection, but laparoscopic resection should be proposed for selected cases in expert centers, depending principally on the tumor size (cut-off 10 cm without any adjacent organ invasion) [21,22]. Therefore, laparoscopic approach should be considered for AONs, as it is safely feasible, especially in the absence of signs for malignity on assessment by imaging and histology like in the present case. Resection involves complete removal of the lesion, without breaching the tumor capsule and with negative resection margins [23]. Panizzo et al. even described a laparoscopic resection for a twelve centimetres oncocytic carcinoma, with good oncological results on follow-up at 24 months [7].

There is no consensus regarding the follow-up after resection and the necessity for an adjuvant treatment [6]. Oncocytic carcinomas however seem to have a better prognosis than other adrenocortical carcinomas [20,24]. The latest systematic review of the literature by Kanitra in 2018 reports a median follow-up of 24 months. Five-year overall survival was 47% for malignant AON, whereas it was 88% for borderline lesions and 100% for benign ones. Survival analyses are however limited by the small number of observations, mostly based on case reports [1].

## 4 Conclusion

AONs are very uncommon tumors with a challenging diagnosis. Laparoscopic resection with clear margins is safe and feasible even in large tumors. There is no consensus in the literature about follow-up after resection and each case should be discussed in a multidisciplinary tumor board.

## Declaration of Competing Interest

The authors report no declarations of interest.

## Funding

There is no extra-founding for this article, for any author.

## Ethical approval

In Switzerland a formal protocol for the EC is not mandatory when less than 5 patients are concerned.

## Consent

The patient was informed about the present article and submission and gave his written consent.

## Author contribution

Penelope St-Amour, Reza Djafarrian and Tobias Zingg wrote the manuscript and contributed together.

Stefano La Rosa reviewed the pathology, produced the pictures of the pathology slides and wrote the corresponding part of the manuscript.

Maurice Matter initiated the article participate to the writing and reviewed the article with Nicolas Demartines.

## Registration of research studies

1. Name of the registry: http://www.researchregistry.com.
2. Unique identifying number or registration ID: researchregistry5960.
3. Hyperlink to your specific registration (must be publicly accessible and will be checked): https://www.researchregistry.com/register-now#home/registrationdetails/5f4904752b51c00018055a1d/.

## Guarantor

Matter M., MD.

## Provenance and peer review

Not commissioned, externally peer-reviewed.
